# Research on missing value prediction of measured ERT data for coal mine based on a GRNN algorithm

**DOI:** 10.1371/journal.pone.0340791

**Published:** 2026-01-13

**Authors:** Pengyu Wang, Xiaofeng Yi, Shumin Wang

**Affiliations:** College of Instrumentation and Electrical Engineering, Jilin University, Changchun, Jilin, China; Henan Polytechnic University, CHINA

## Abstract

In the process of long-term monitoring of the coal seam floor of a coal mining face using electrical resistivity tomography (ERT), the data loss caused by electrode disconnection adversely affects early warning of water inrush and prevents the identification of hidden dangers, hindering safe production. Due to the particularity of the monitored environment, the maintenance of offline electrodes may not be timely. Therefore, how to deal with the loss of measured data caused by electrode disconnection has become a problem that must be solved in the long-term monitoring process. In this paper, we analyze the effect of electrode disconnection on the measured data. Then, the principle of the general regression neural network (GRNN) algorithm is introduced. The missing values in the measured data are predicted using the GRNN algorithm. The results of verification experiments conducted in a water tank show that when the original data integrity is 82.96%, the predicted data accuracy reaches 91.46%, and when the original data integrity is only 55.56%, the predicted data accuracy still reaches 82.45%. Finally, actual applications of the proposed method are carried out on coal mining faces. A set of data with an integrity of 73.8% is predicted. Compared with the measured data when all the electrodes are online, the accuracy of the predicted data is 85.18%. The accuracy of the data predicted using the proposed method is 14.99% higher than that of the data predicted using the commonly used mean value interpolation method.

## Introduction

Coal is an important basic energy source and raw material, and it plays an important role in economic development [1]. China is currently the largest coal producer in the world [2], and it is also one of the countries that are most seriously affected by water inrush during coal mining [3]. Therefore, determining the location of water-bearing media in the coal seam floor is of great significance to the safe production of coal mines, and electrical resistivity tomography (ERT), which is sensitive to low-resistivity anomalous bodies such as water, has unique advantages [4]. It has been used in geothermal monitoring [5], volcanology studies [6], infrastructure stability [7], landslide monitoring [8], permafrost monitoring [9], archaeology [10], and other fields, and it has achieved good results.

With the development of ERT technology, this method has been used to realize long-term monitoring of changes in electrical information of the coal seam floor. In the long-term monitoring process, the equipment is placed on the coal mining face for a long time. However, due to the complex environment of the coal mining face, the coupling between the electrodes and the floor cannot be guaranteed; that is, factors such as vibration during the coal mining process and unintentional damage to the electrode cable caused by construction machinery may cause the electrodes to be offline. Electrode disconnection will lead to partial loss of measured data, and improper handling of the missing values will cause relevant valuable information to be ignored [11], which affects the subsequent data analysis, leads to deviations in the data analysis results, and even causes false and missed alarms in water inrush early warning.

In research on the prediction of missing values in measured data related to coal mines, Ru et al. [12] proposed that the correlation coefficient can be used to predict missing values in measured data of coal and gas outburst. Song et al. [13] proposed that the lasso regression algorithm can be used to predict the missing values in measured data of the gas concentration. Shao et al. [14] proposed that the random forest prediction model can be used to predict missing values in measured data of the coal and gas outburst. However, the above missing value prediction methods are all aimed at measured data of gas, and little research has been conducted on the prediction of missing values in measured ERT data. Currently, the main method for dealing with missing ERT values is the mean value interpolation method, but this method has great limitations. First, the mean value interpolation method is not suitable for the case of an uneven data distribution. However, there are various media in the coal seam floor, so the measured data are usually not evenly distributed, and the predicted data may not be accurate. Second, the mean value interpolation method is only applicable when there are a sufficient number of known points around the interpolation point. However, when electrodes are continuously offline, the missing values usually appear continuously, so the predicted data may not be reliable. Finally, the mean value interpolation method only considers the average of the known values, and it does not consider the correlation of the original data [15–17].

Because of the limitations of the mean value interpolation method, we aim to find a more accurate and reliable missing value prediction method. Neural networks have been a research hotspot in the field of artificial intelligence since the 1980s [18]. With the gradual deepening of research on neural networks, researchers have proposed a series of models, such as the backpropagation neural network (BPNN) and radial basis function neural network (RBFNN). Specht proposed the general regression neural network (GRNN) in 1991 [19]. Compared with the BPNN and RBFNN models, the GRNN model has the following advantages: (1) It has fewer model parameters. Only the smoothing parameter needs to be considered in the GRNN model, so many optimization steps can be omitted. In contrast, parameters such as the weight and bias need to be considered in the BPNN model, and parameters such as the width and center position of the basis function need to be considered in the RBFNN model. It makes the GRNN model simpler and more intuitive in terms of the parameter setting, which reduces the influence of human factors on the predicted results. (2) It has a fast convergence rate. Since there is no iteration in the training process, the GRNN model has a faster convergence rate than the BPNN and RBFNN models. (3) It has a strong generalization ability. The GRNN model is especially suitable for small sample regression problems, while the BPNN and RBFNN models are not as stable as the GRNN model when processing small sample data [20–23]. Based on the above advantages, the GRNN model has been widely applied in pattern recognition, information prediction, and other fields [24–25].

When ERT is used to conduct long-term monitoring of a coal seam floor, to ensure the attainment of real-time monitoring and early warning abilities, the algorithm used to conduct the data processing requires a faster convergence speed. In addition, due to the small sample size of single measured data, the algorithm used to conduct the data processing requires a stronger generalization ability. Therefore, based on the above engineering characteristics, the GRNN model has more obvious rationality and superiority compared with the BPNN and RBFNN models.

In summary, the goal of this study is to address the problem of the loss of measured data caused by electrode disconnection, and the GRNN algorithm is determined to be very suitable for achieving this goal. This method eliminates the need for engineers to go to the coal mining face to maintain the offline electrodes at unscheduled times during the long-term monitoring process, reducing the measurement cost. It can also reduce the possibility of false and missed alarms caused by missing data and can better guarantee the realization of safe production.

## Materials and methods

Before predicting the missing values, it is necessary to analyze what impact offline electrodes will exert on the measured data and how large the impact range will be. Next, the analysis starts from the principle of ERT.

**Fig 1:**
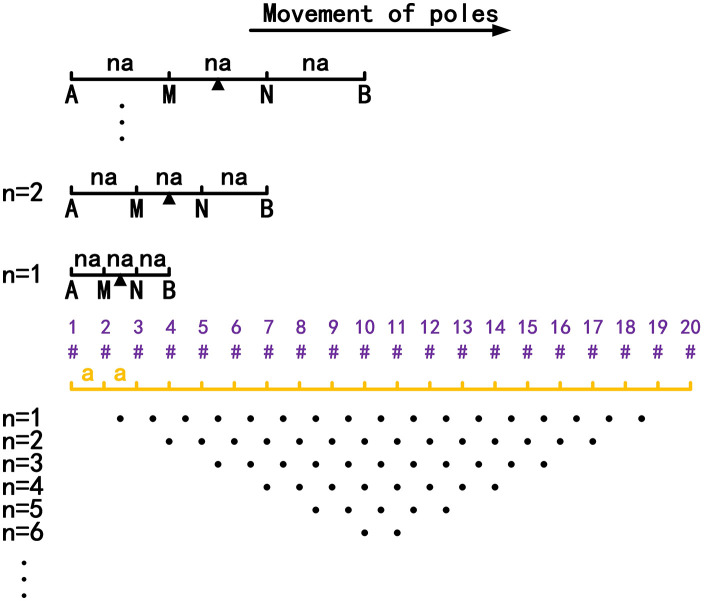
Working principle of a Wenner α array.


A Wenner α array is taken as an example to illustrate the working principle of ERT, as shown in Fig 1 [26]. In the apparent resistivity profile, the method for calculating the apparent resistivity $\rho_{a}$ of each measured point is as follows:

$$\rho_{a}=K_{\alpha }\cdot \frac{\Delta U_{MN}}{I_{AB}}$$ (1)

where $\Delta U_{MN}$ is the potential difference between potential electrodes M and N, $I_{AB}$ is the current between current electrodes A and B, and $K_{\alpha }$ is the geometric factor, which is a quantity that only depends on the locations of the poles. The geometric factor $K_{\alpha }$ can be expressed as follows:


$$K_{\alpha }=\frac{2\pi }{\frac{\text{1}}{\left|AM\right|}-\frac{\text{1}}{\left|AN\right|}-\frac{\text{1}}{\left|BM\right|}+\frac{\text{1}}{\left|BN\right|}}$$ (2)

As can be seen from Fig 1, when any electrode is offline, if it acts as the current pole, $I_{AB}$ will theoretically approach 0. In contrast, if it acts as the potential pole, $\Delta U_{MN}$ will theoretically approach 0. Therefore, when the offline electrode acts as current pole A or B, by substituting $I_{AB}\to 0$ into Eq. (1), we obtain $\rho_{a}\to \infty$, and the corresponding measured points are the high-resistivity anomalous points in the apparent resistivity contour map. In contrast, when the offline electrode acts as potential pole M or N, by substituting $\Delta U_{MN}\to 0$ into Eq. (1), we obtain $\rho_{a}\to 0$, and the corresponding measured points are the low-resistivity anomalous points in the apparent resistivity contour map.

**Fig 2:**
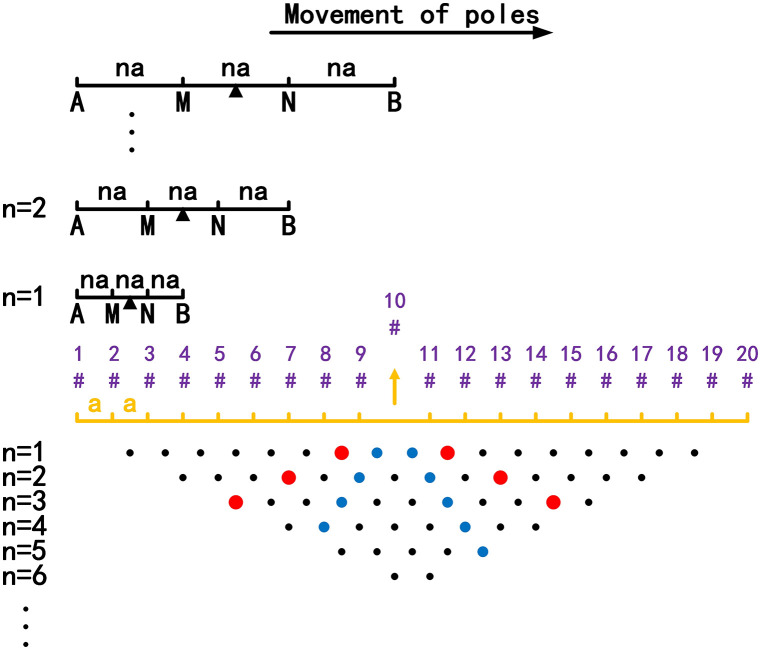
Apparent resistivity profile: Any electrode is offline. The blue points are the low-resistivity anomalous points ($\rho_{a}\to 0$), and the red points are the high-resistivity anomalous points ($\rho_{a}\to \infty$). The distribution of the anomalous points is radial.

From the above analysis, it can be seen that when any electrode in Fig 1 is offline, the obtained apparent resistivity profile is the same as that shown in Fig 2, and the distribution of the anomalous points is radial. If an anomalous point is deleted, the data corresponding to this anomalous point is a missing value. Currently, the commonly used method for dealing with missing ERT values is the mean value interpolation method. However, when the data distribution is not uniform or the missing values appear continuously, this method will destroy the correlation of the original data, resulting in inaccurate predicted results. Therefore, to solve the above problems, in our proposed method, the missing values are predicted using the GRNN algorithm.

**Fig 3:**
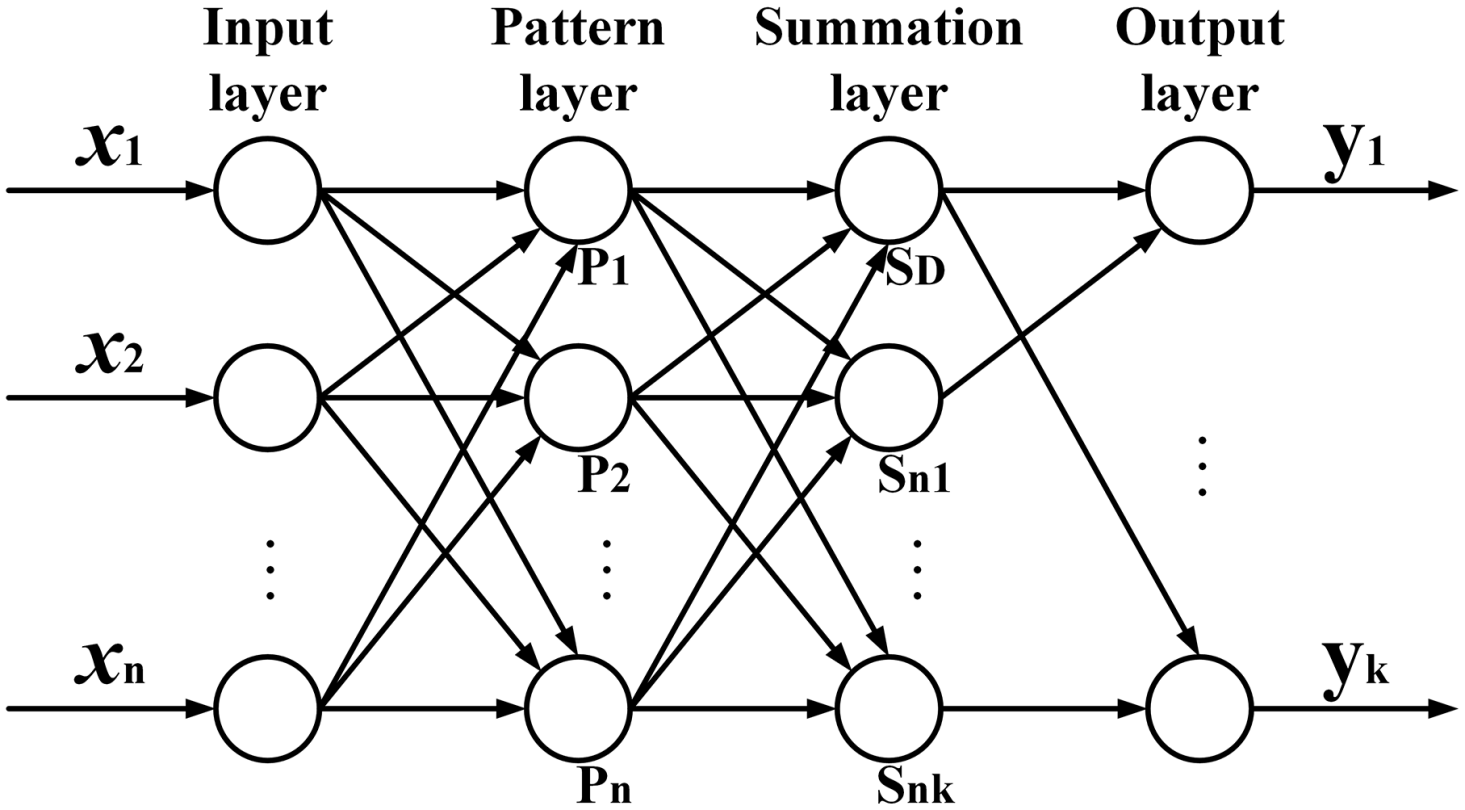
Network structure of GRNN.

The GRNN model is composed of four layers, namely, input layer, pattern layer, summation layer, and output layer, as shown in Fig 3.

The input is $𝐗={\left[x_{1},x_{2},\cdots ,x_{n}\right]}^{T}$, and the output is $𝐘={\left[y_{1},y_{2},\cdots ,y_{k}\right]}^{T}$. The number of input layer nodes is equal to the dimension of the input vector, and each node transfers the input sample directly to the pattern layer. The transfer function of the pattern layer is the radial basis function, which is calculated as follows:


$$P_{i}=\exp\left[-\frac{{\left(𝐗-{𝐗}_{i}\right)}^{T}\left(𝐗-{𝐗}_{i}\right)}{2\sigma^{2}}\right]$$ (3)

where **X** is the input variable, ${𝐗}_{i}$ is the learning sample corresponding to the ith neuron, and σ is the sample probability of width, i.e., the smoothing parameter.

Two types of summation functions are used in the summation layer to sum the outputs of the pattern layer. The first type of summation is $\sum_{i=1}^{n}\exp\left[-\frac{{\left(𝐗-{𝐗}_{i}\right)}^{T}\left(𝐗-{𝐗}_{i}\right)}{2\sigma^{2}}\right]$, which represents the sum of all the outputs in the pattern layer. The connection weight between the pattern layer node and the summation layer node is 1, so the transfer function is calculated as follows:


$$S_{D}=\sum_{i=1}^{n}P_{i}$$ (4)

The second type of summation is $\sum_{i=1}^{n}{𝐘}_{i}\exp\left[-\frac{{\left(𝐗-{𝐗}_{i}\right)}^{T}\left(𝐗-{𝐗}_{i}\right)}{2\sigma^{2}}\right]$, which represents the weighted sum of the expected outputs and the outputs of the pattern layer nodes. The connection weight between the pattern layer node and the summation layer node is the expected output data, namely, the jth element in the ith output sample ${𝐘}_{i}$, so the transfer function is calculated as follows:


$$S_{nj}=\sum_{i=1}^{n}y_{ij}P_{i}$$ (5)

The number of output layer nodes is equal to the dimension of the output vector. The output value can be obtained by dividing the output of the summation layer of the second type by that of the first type, which is calculated as follows:


$$y_{j}=\frac{S_{nj}}{S_{D}}$$ (6)

The theoretical basis of the GRNN model is nonlinear regression [27]. The joint probability density function f(𝐱,y) of a random vector variable **x** (independent variable) and a random scalar variable y (dependent variable) can be obtained from the observed sample, so as to obtain the regression of the dependent variable on the independent variable. Given that the observed value of **x** is **X**, the regression of y on **X** (i.e., the conditional mean of y given **X**) is:


$$E\left(y|𝐗\right)=\frac{\int_{-\infty }^{+\infty }yf\left(𝐗,y\right)dy}{\int_{-\infty }^{+\infty }f\left(𝐗,y\right)dy}$$ (7)

When f(𝐱,y) is not known, it can be estimated from the sample observations of **x** and y, and the probability estimator $\hat{f}(𝐗,Y)$ is calculated as follows:


$$\hat{f}(𝐗,Y)=\frac{\text{1}}{{\left(2\pi \right)}^{\left(\frac{p+1}{\text{2}}\right)}\sigma^{\left(p+1\right)}}\cdot \frac{\text{1}}{n}\sum_{i=1}^{n}\exp\left[-\frac{{\left(𝐗-{𝐗}^{i}\right)}^{T}\left(𝐗-{𝐗}^{i}\right)}{2\sigma^{2}}\right]\cdot \exp\left[-\frac{{\left(Y-Y^{i}\right)}^{2}}{2\sigma^{2}}\right]$$ (8)

where p is the dimension of the vector variable **x**, n is the number of sample observations, ${𝐗}^{i}$ is the sample observation of **x**, and $Y^{i}$ is the sample observation of y.

By substituting Eq. (8) into Eq. (7), we can obtain:


$$\hat{Y}\left(𝐗\right)=\frac{\sum_{i=1}^{n}\exp\left[-\frac{{\left(𝐗-{𝐗}^{i}\right)}^{T}\left(𝐗-{𝐗}^{i}\right)}{2\sigma^{2}}\right]\int_{-\infty }^{+\infty }y\exp\left[-\frac{{\left(y-Y^{i}\right)}^{2}}{2\sigma^{2}}\right]dy}{\sum_{i=1}^{n}\exp\left[-\frac{{\left(𝐗-{𝐗}^{i}\right)}^{T}\left(𝐗-{𝐗}^{i}\right)}{2\sigma^{2}}\right]\int_{-\infty }^{+\infty }\exp\left[-\frac{{\left(y-Y^{i}\right)}^{2}}{2\sigma^{2}}\right]dy}$$ (9)

The scalar function $D_{i}^{2}$ is defined as follows:


$$D_{i}^{2}={\left(𝐗-{𝐗}^{i}\right)}^{T}\left(𝐗-{𝐗}^{i}\right)$$ (10)

By substituting Eq. (10) into Eq. (9), we can obtain


$$\hat{Y}\left(𝐗\right)=\frac{\sum_{i=1}^{n}Y^{i}\exp\left(-\frac{D_{i}^{2}}{2\sigma^{2}}\right)}{\sum_{i=1}^{n}\exp\left(-\frac{D_{i}^{2}}{2\sigma^{2}}\right)}$$ (11)

The above calculation is the basic principle of the GRNN model. As can be seen from Eq. (11), setting the parameter of the GRNN model is convenient, and the performance of the GRNN model can be improved by setting the smoothing parameter σ in the kernel function. In addition, unlike conventional neural networks, the GRNN model does not need to be iteratively trained [28].

When the GRNN model is used to predict missing values in measured ERT data, the abscissa and ordinate of each measured point are used to form two data dimensions, and the parameter set $𝐗=\left\{{𝐱}_{1},{𝐱}_{2}\right\}$ is established. In the parameter set, x is taken as the input of the GRNN network, and the apparent resistivity value y of the corresponding measured point is used as the output. Thus, the mapping relationship between **X** and y is established. Assuming that there are m measured points in a measured apparent resistivity profile, including n missing values, the original data integrity is $\frac{m-n}{m}$. In the prediction process, ${\left\{x_{1,1},x_{1,2},\cdots ,x_{1,m-n}\right\}}^{T}$, ${\left\{x_{2,1},x_{2,2},\cdots ,x_{2,m-n}\right\}}^{T}$, and ${\left\{y_{1},y_{2},\cdots ,y_{m-n}\right\}}^{T}$ are used as the training set; and ${\left\{x_{1,m-n+1},x_{1,m-n+2},\cdots ,x_{1,m}\right\}}^{T}$, ${\left\{x_{2,m-n+1},x_{2,m-n+2},\cdots ,x_{2,m}\right\}}^{T}$, and ${\left\{y_{m-n+1},y_{m-n+2},\cdots ,y_{m}\right\}}^{T}$ are used as the testing set.

For the data in the testing set, the quality of the prediction can be judged by calculating the error between the predicted values and the true values.

## Results

### Experiments in a water tank

Firstly, the experiments are carried out in a plastic water tank. The length of the water tank is 2 m, the width is 1 m, and the height is 1 m. For ease of operation, the survey line length should not exceed 1.5 m. The selection of the electrode spacing a is constrained by the survey line length L and the number of electrodes N, and a is calculated as follows:


$$a=\frac{L}{N-1}$$ (12)

As can be seen from Fig 1, the larger N is, the larger the number of measured points is. Therefore, to obtain as many measured points as possible, the electrode spacing should be as small as possible when the survey line length is determined. However, to avoid the occurrence of columnar discharge, the water entry depth D is usually calculated as follows:


$$D\leq \frac{a}{10}$$ (13)

As can be seen from Eq. (13), the water entry depth should be as small as possible, but too small a water entry depth will lead to poor contact between the electrode and the water surface. Taking the above factors into consideration, it is finally determined that the water entry depth, electrode spacing, and number of electrodes should be 0.5 cm, 5 cm, and 30, respectively.

When the size of the anomalous body is too small, the change of the apparent resistivity of adjacent measured points is not obvious, so the data distribution is relatively uniform, and the processing of such data cannot better demonstrate the superiority of the GRNN model compared with conventional methods. Therefore, on the premise of determining the electrode spacing of 5 cm, the length of the used low-resistivity anomalous body is set to 23 cm, the height is set to 15 cm, and the water surface height is set to 20 cm.

**Fig 4:**
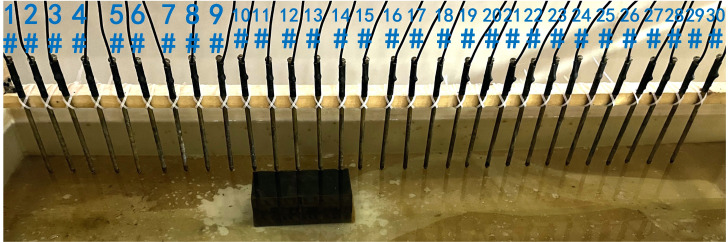
Verification experiment: The layout of the experiment. All the electrodes are online.

**Fig 5:**
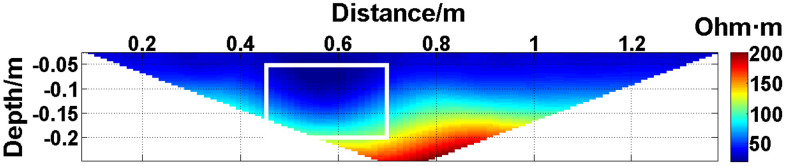
Verification experiment: The measured apparent resistivity contour map. The low-resistivity area marked by the white box is basically consistent with the location of the iron block in Fig 4.

In summary, when all the electrodes are online, the layout of the experiment is shown in Fig 4. The material of the electrodes is brass, and 30 electrodes are fixed vertically downward on a square wood beam with an electrode spacing of 5 cm. The water entry depth of the tips is the same (approximately 0.5 cm) and can be regarded as a point contact. The low-resistivity anomalous body is an iron block with a length of 23 cm and a height of 15 cm. The anomalous body is located below electrodes #10-#14 and is completely submerged in the water. The array type is set as a Wenner α array. The measured apparent resistivity contour map is shown in Fig 5, and the low-resistivity area is basically consistent with the location of the iron block shown in Fig 4.

**Fig 6:**
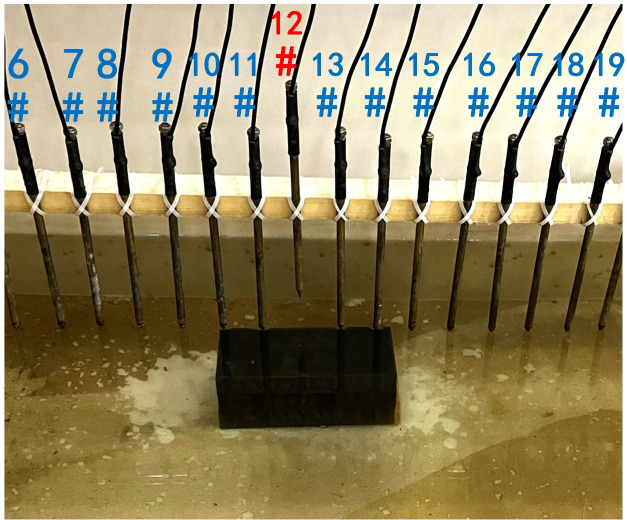
Verification experiment: One electrode is offline.

**Fig 7:**
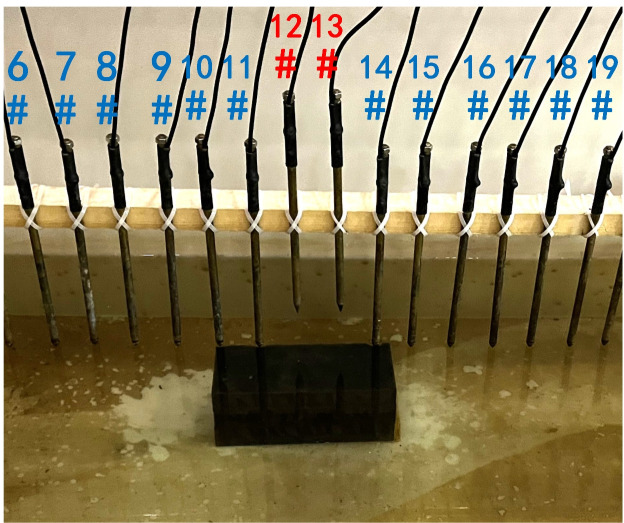
Verification experiment: Two electrodes are continuously offline.

**Fig 8:**
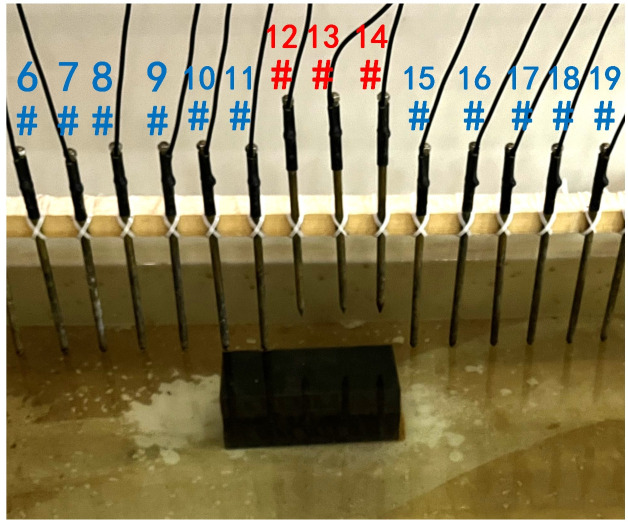
Verification experiment: Three electrodes are continuously offline.

**Fig 9:**
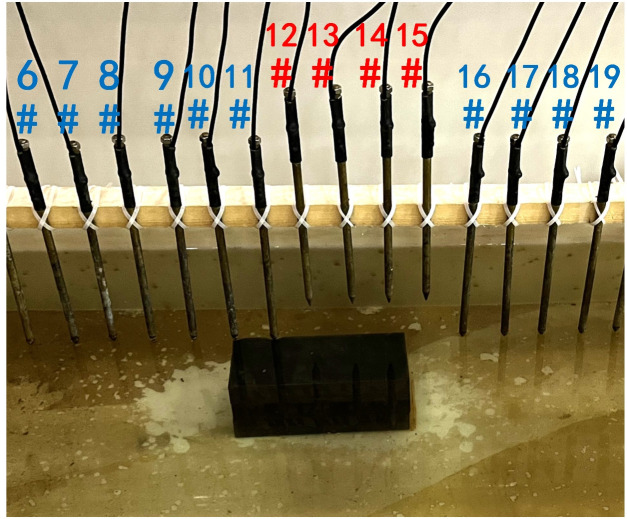
Verification experiment: Four electrodes are continuously offline.

**Fig 10:**
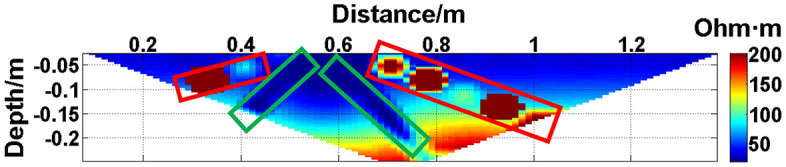
Apparent resistivity contour map: One electrode is offline. The areas marked by the red boxes are the high-resistivity anomalous points, and the areas marked by the green boxes are the low-resistivity anomalous points. The distribution of the anomalous points is radial, which is consistent with Fig 2.

**Fig 11:**
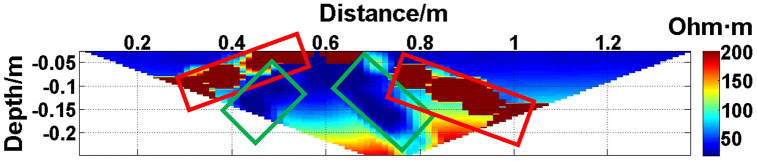
Apparent resistivity contour map: Two electrodes are continuously offline.

**Fig 12:**
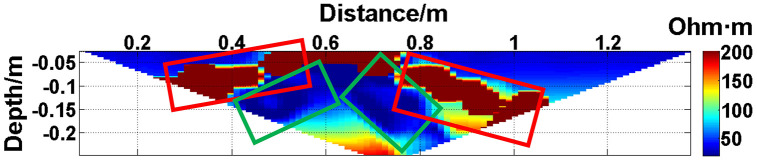
Apparent resistivity contour map: Three electrodes are continuously offline.

**Fig 13:**
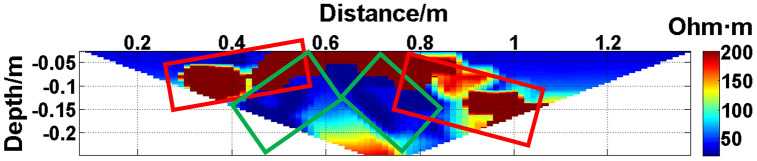
Apparent resistivity contour map: Four electrodes are continuously offline.

To verify the effect of electrode disconnection on the measured data, 1, 2, 3, and 4 electrodes are lifted up to leave the water surface successively, and the electrodes are no longer in contact with the water surface. To make the correlation of the original data as broken as possible, the offline electrodes are continuous, as shown in Figs 6–9. The array type is also set as a Wenner α array, and the measured apparent resistivity contour maps are shown in Figs 10–13. The distribution of the anomalous points is radial, which is consistent with Fig 2. As the number of offline electrodes increases, the number of anomalous points gradually increases, and the effect on the apparent resistivity contour map also gradually increases.

The apparent resistivity profile shown in Fig 10 contains a total of 135 measured points, including 23 anomalous points. By deleting the 23 corresponding values, a set of data containing 23 missing values is obtained, and the original data integrity is 82.96%.

As can be seen from Eq. (11), the choice of the smoothing parameter has a great influence on the fitting ability of the GRNN model. Too small a σ value will lead to overfitting, while too large a σ value will lead to underfitting. Therefore, to obtain the best prediction, the value of σ is determined by the K-fold cross-validation, and K is set as 4. The value range of σ is [0.1, 2], and the step size is set as 0.1. After four cross validations, the best σ value is determined to be 0.1.

**Fig 14:**
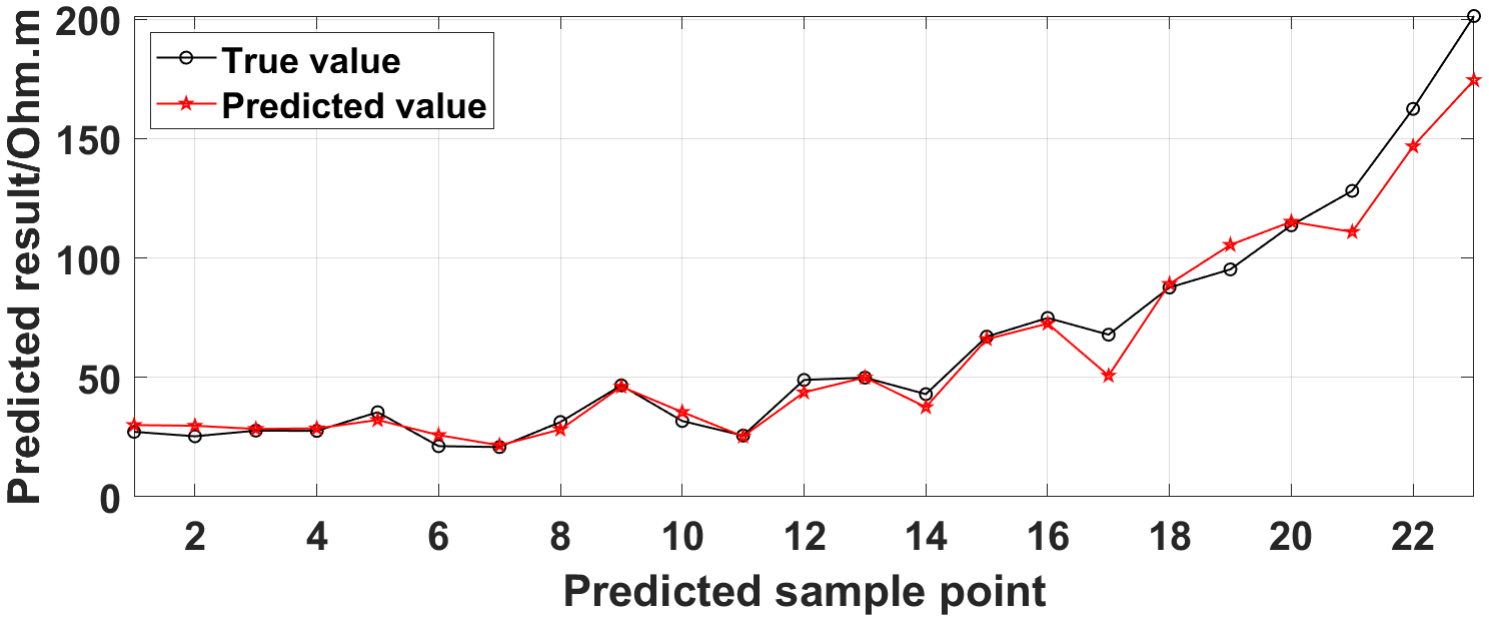
Comparison of the predicted values and true values: One electrode is offline.

The GRNN algorithm is used to process the data presented in Fig 10, and a comparison between the predicted values and true values is shown in Fig 14.

**Fig 15:**
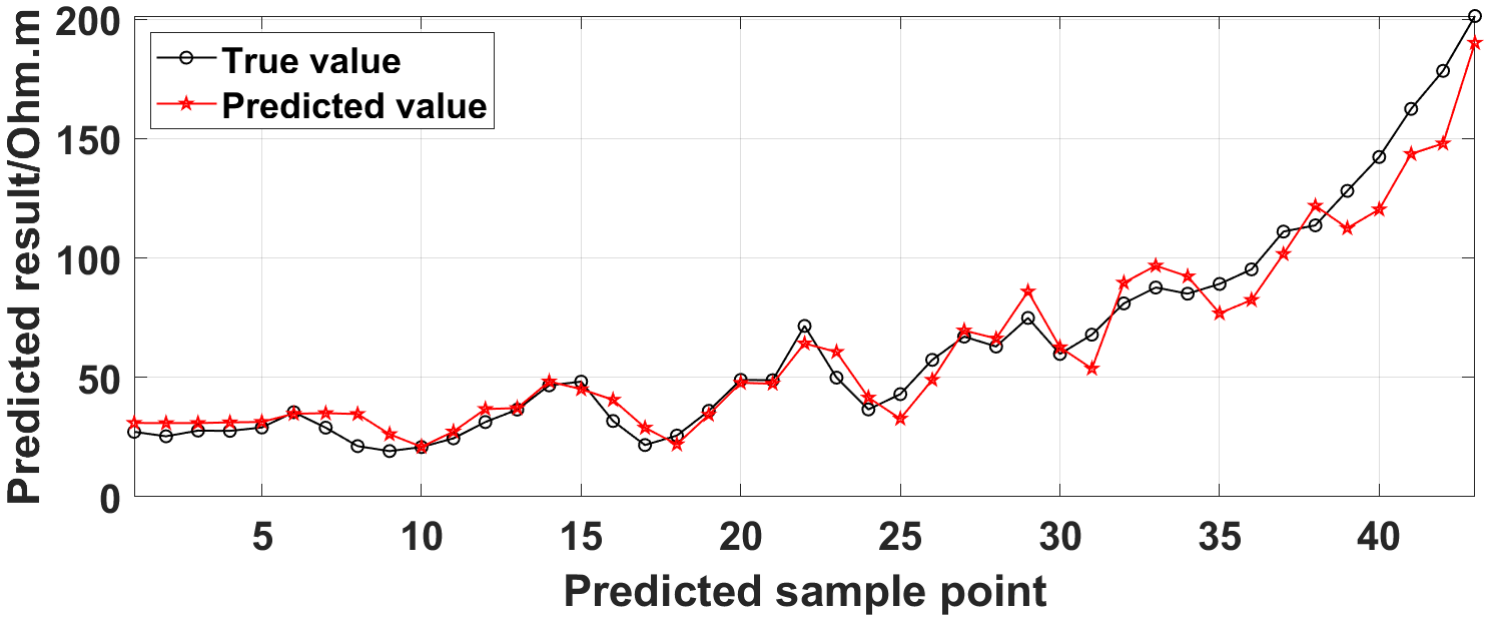
Comparison of the predicted values and true values: Two electrodes are continuously offline.

**Fig 16:**
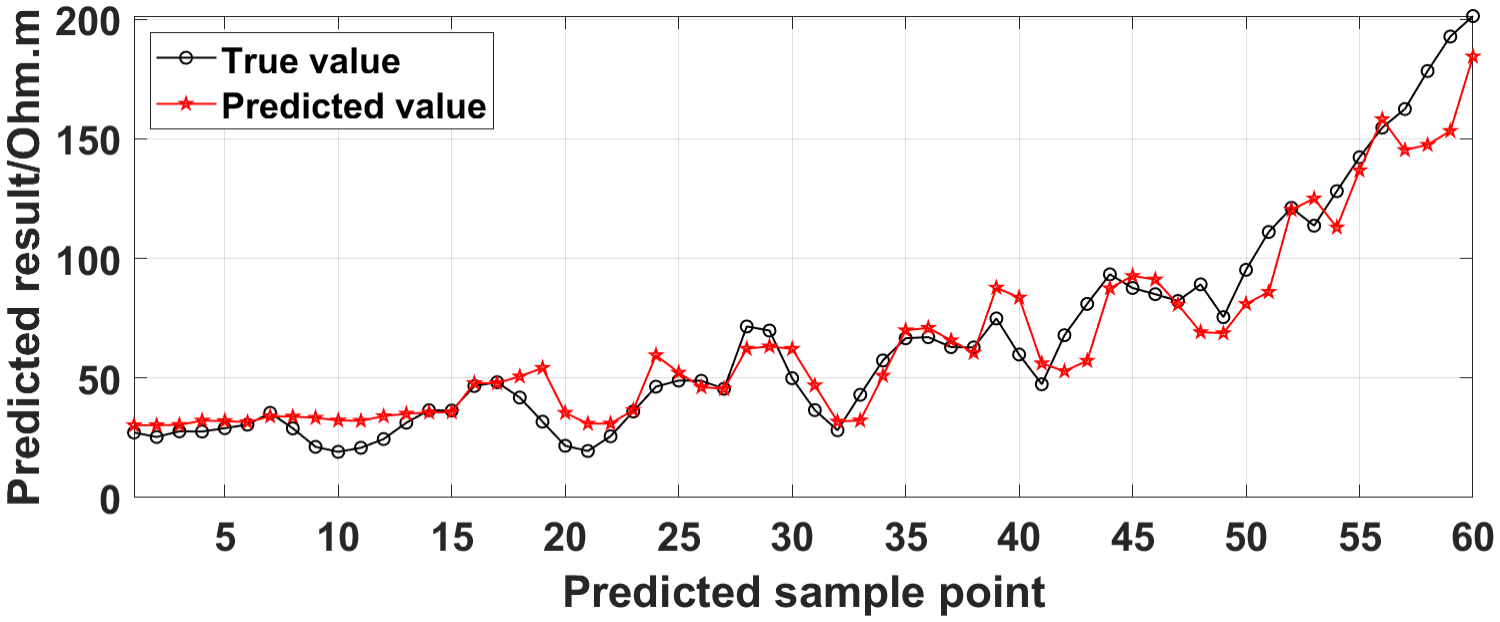
Comparison of the predicted values and true values: Three electrodes are continuously offline.

**Fig 17:**
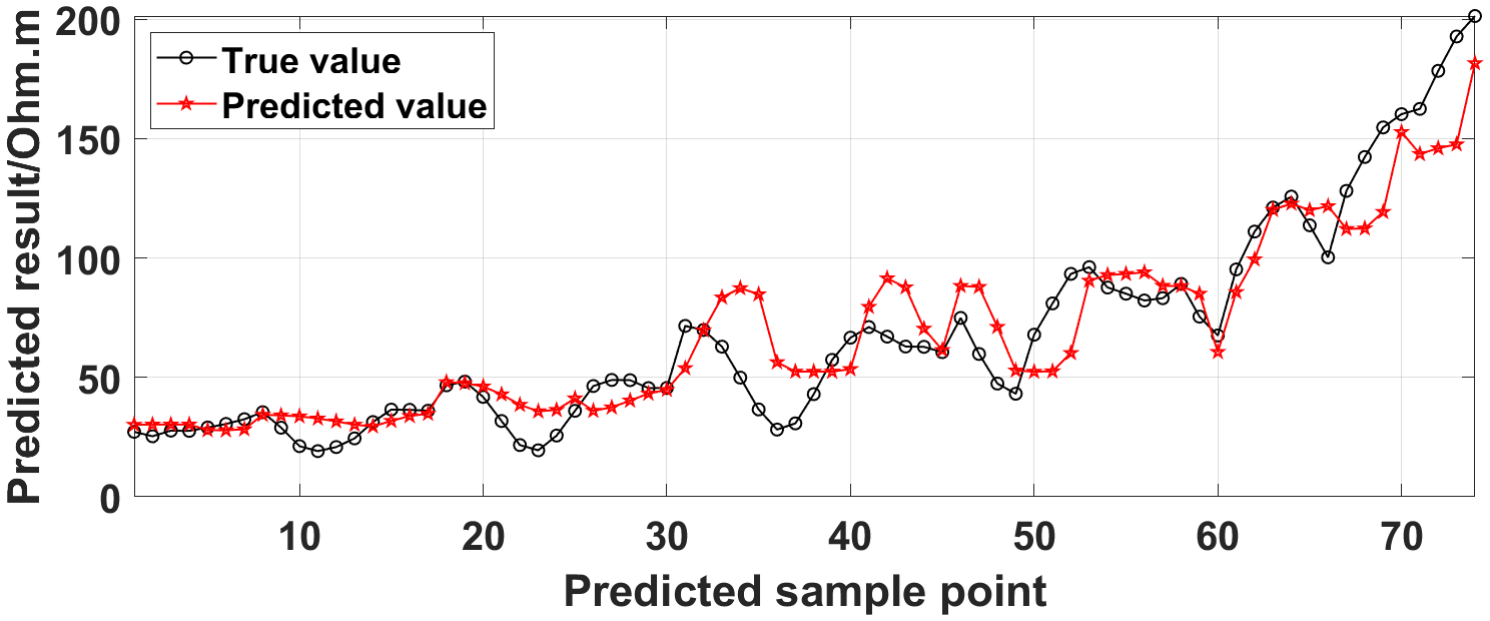
Comparison of the predicted values and true values: Four electrodes are continuously offline.

Similarly, the apparent resistivity profile shown in Fig 11 contains 43 anomalous points, and the original data integrity is 68.15%. The apparent resistivity profile shown in Fig 12 contains 60 anomalous points, and the original data integrity is 55.56%. The apparent resistivity profile shown in Fig 13 contains 74 anomalous points, and the original data integrity is 45.19%. The above three groups of data are processed using the GRNN algorithm, and comparisons of the corresponding predicted values and true values are shown in Figs 15–17.

The missing values in the original data in Figs 10–13 are filled with the predicted values, and the obtained apparent resistivity contour maps are shown in Figs 18–21.

**Fig 18:**
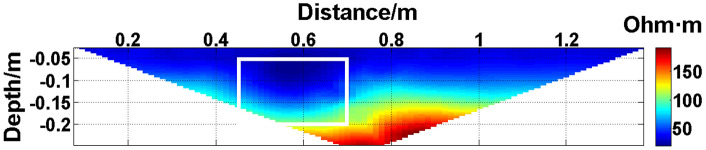
Apparent resistivity contour map obtained by filling in the missing values in the original data in Fig 10 with the predicted values: One electrode is offline. The area marked by the white box corresponds to the location of the iron block in Fig 4.

**Fig 19:**
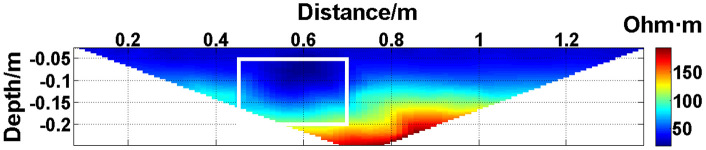
Apparent resistivity contour map obtained by filling in the missing values in the original data in Fig 11 with the predicted values: Two electrodes are continuously offline.

**Fig 20:**
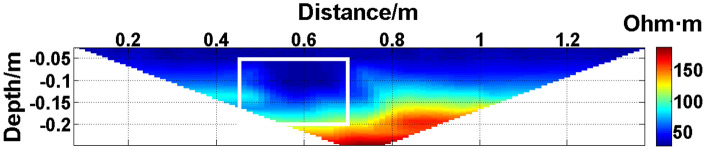
Apparent resistivity contour map obtained by filling in the missing values in the original data in Fig 12 with the predicted values: Three electrodes are continuously offline.

**Fig 21:**
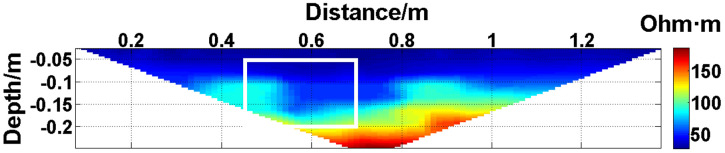
Apparent resistivity contour map obtained by filling in the missing values in the original data in Fig 13 with the predicted values: Four electrodes are continuously offline.

**Fig 22:**
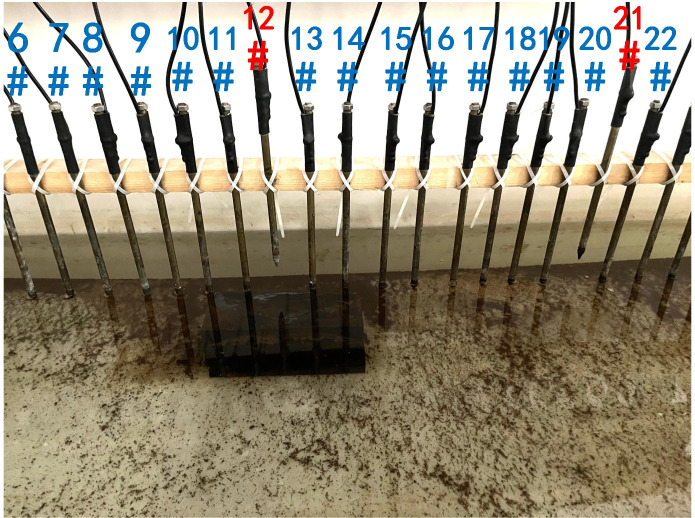
Verification experiment of random electrode disconnection: Electrodes #12 and #21 are offline.

**Fig 23:**
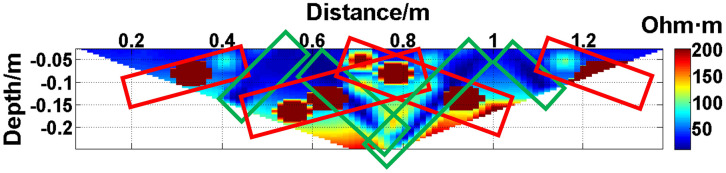
Apparent resistivity contour map of Fig 22: The distribution of anomalous points is radial centered on electrodes #12 and #21.

Next, the case of random electrode disconnection is simulated, and the layout of the experiment is shown in Fig 22. Electrodes #12 and #21 are lifted up to leave the water surface, so the electrodes are no longer in contact with the water surface. The array type is also set as a Wenner α array, and the measured apparent resistivity contour map is shown in Fig 23. The distribution of the anomalous points is radial and is centered on electrodes #12 and #21.

**Fig 24:**
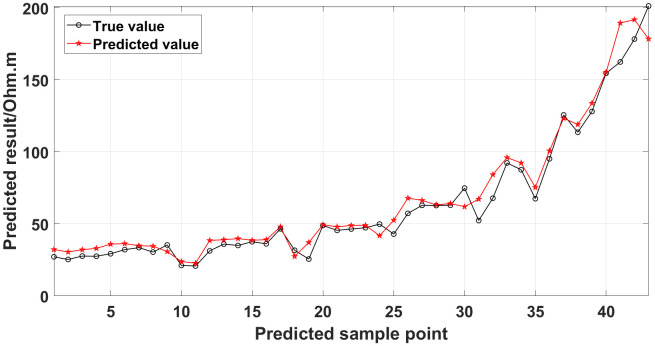
Comparison of the predicted values and true values: Electrodes are randomly offline.

The apparent resistivity profile shown in Fig 23 contains a total of 135 measured points, including 43 anomalous points. By deleting the 43 corresponding values, a set of data containing 43 missing values is obtained, and the original data integrity is 68.15%. The GRNN algorithm is used to process the data, and a comparison between the predicted values and true values is shown in Fig 24.

**Fig 25:**
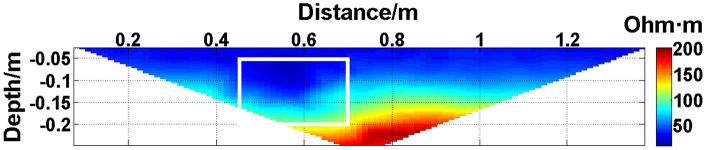
Apparent resistivity contour map obtained by filling in the missing values in the original data in Fig 23 with the predicted values.

The missing values in the original data in Fig 23 are filled with the predicted values, and the obtained apparent resistivity contour map is shown in Fig 25.

### Applications in coal mining faces

To demonstrate the generalizability of the method proposed in this paper, case studies of different coal mining faces are selected, and the electrode disconnection modes are also different.

**Fig 26:**
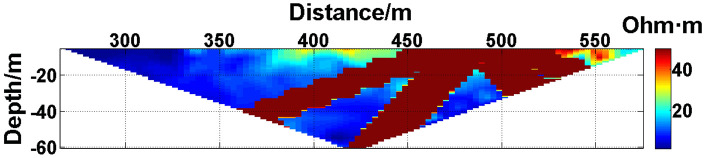
Apparent resistivity contour map of the coal seam floor of the 1295 coal mining face of the Gequan East Mine on October 13, 2021: Electrodes #49 and #50 are continuously offline.

**Fig 27:**
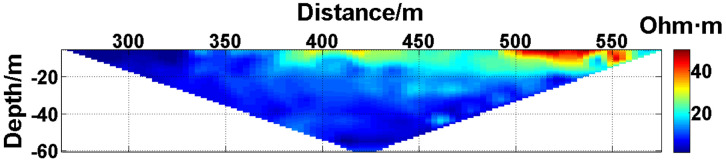
Apparent resistivity contour map of the coal seam floor of the 1295 coal mining face of the Gequan East Mine on October 12, 2021: All the electrodes are online.

**Fig 28:**
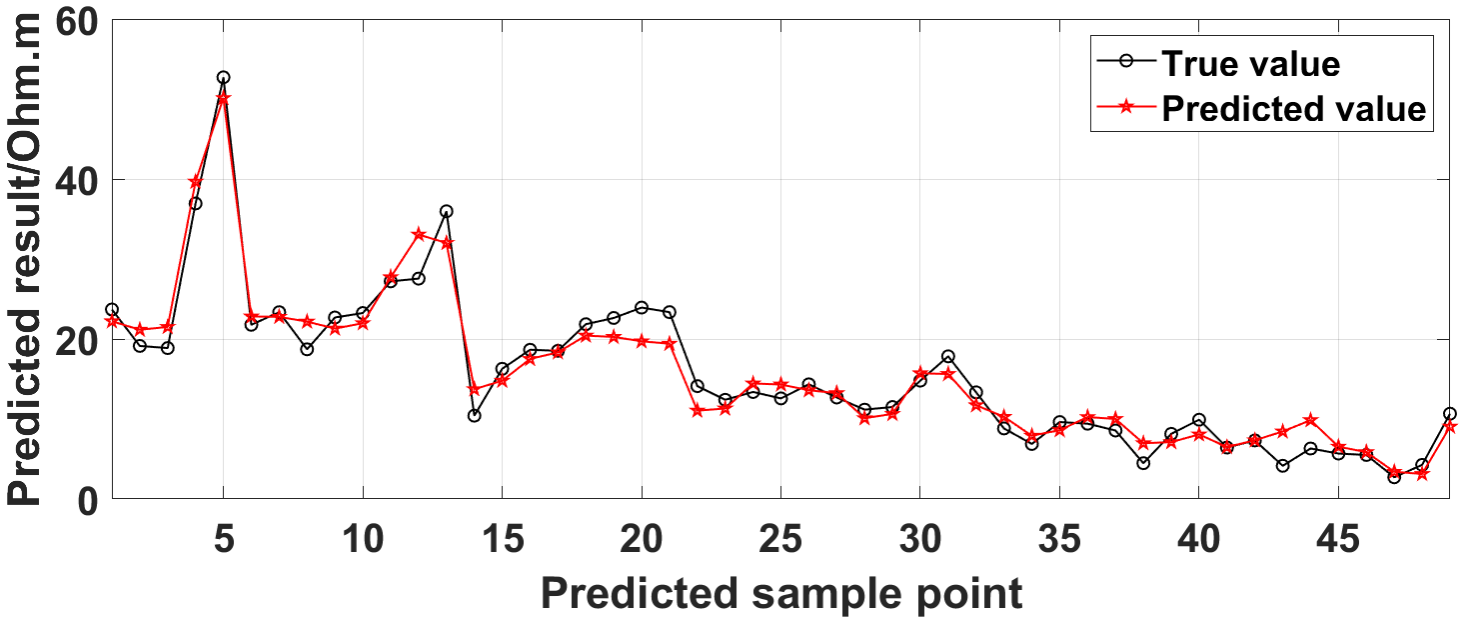
Comparison of the predicted values of the missing values in Fig 26 (October 13, 2021) and the true values at the corresponding measured points in Fig 27 (October 12, 2021).

**Fig 29:**
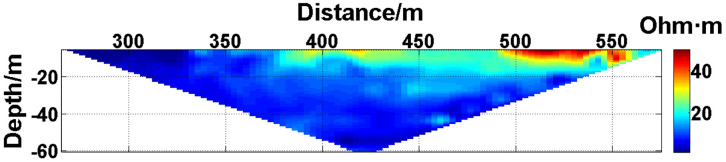
Apparent resistivity contour map obtained by filling in the missing values in the original data (October 13, 2021) with the predicted values.

**Fig 30:**
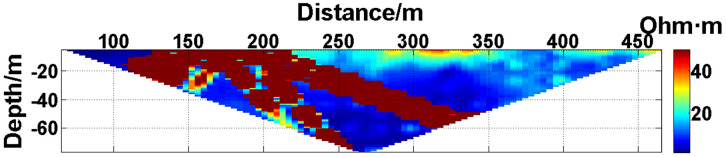
Apparent resistivity contour map of the coal seam floor of the Ji 17-33200 coal mining face of the Pingdingshan #10 Mine on September 15, 2022: Electrodes #17 and #19 are discontinuously offline.

**Fig 31:**
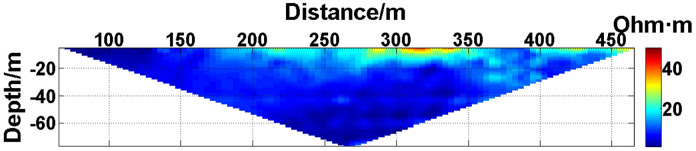
Apparent resistivity contour map of the coal seam floor of the Ji 17-33200 coal mining face of the Pingdingshan #10 Mine on September 14, 2022: All the electrodes are online.

**Fig 32:**
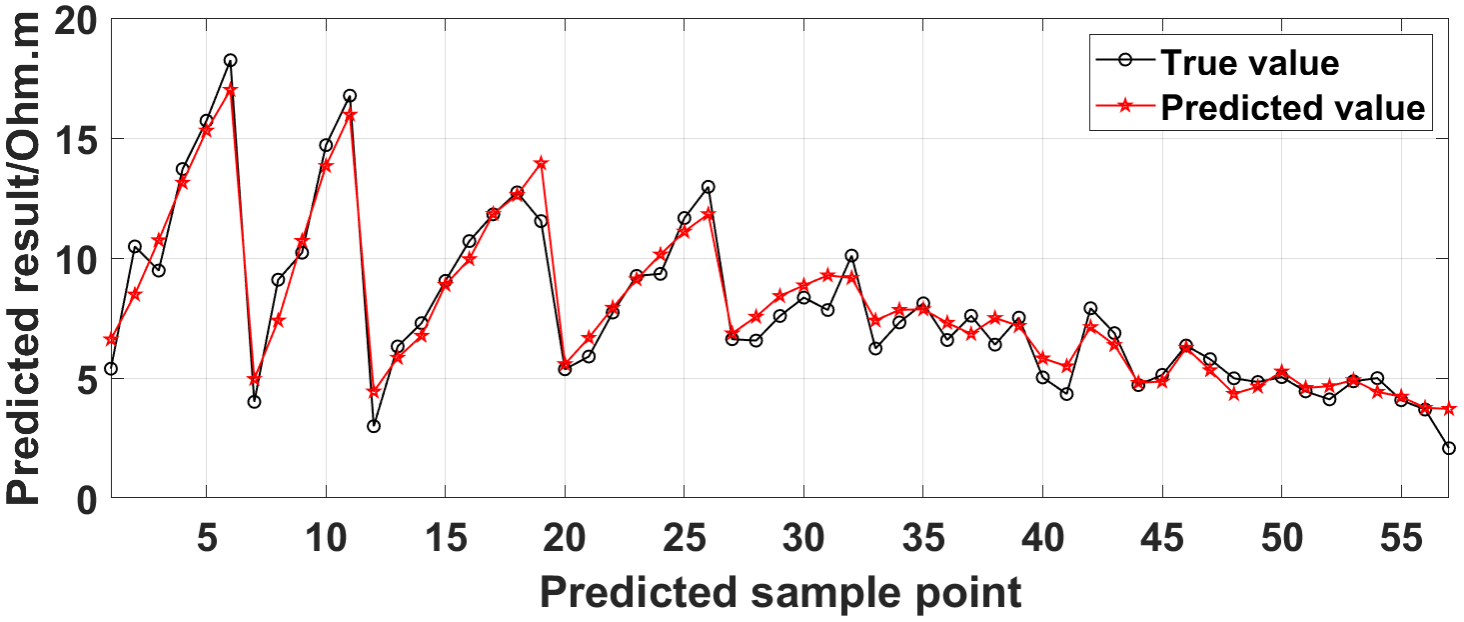
Comparison of the predicted values of the missing values in Fig 30 (September 15, 2022) and the true values at the corresponding measured points in Fig 31 (September 14, 2022).

**Fig 33:**
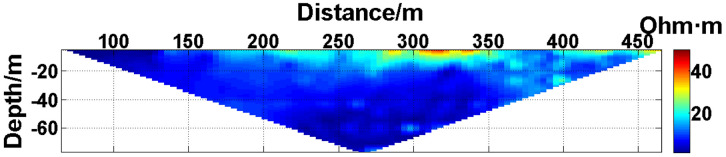
Apparent resistivity contour map obtained by filling in the missing values in the original data (September 15, 2022) with the predicted values.

(1) Continuous electrode disconnection mode: This case is from the 1295 coal mining face of the Gequan East Mine of the Jizhong Energy Co., Ltd., which is located in Xingtai City, Hebei Province, China. A total of 60 brass electrodes with lengths of 0.25 m and diameters of 0.01 m are laid in the roadway at one side of the coal mining face, and electrodes #26 to #60 are located in the effective monitoring zone. They are laid at the junction of the coal seam and its floor along the strike direction, and the electrode spacing is 10 m. To ensure good coupling between the electrodes and the coal seam floor, the electrodes are completely inserted into the coal seam floor by hammering, and the gaps are filled with yellow mud. The array type is set as a Wenner α array. The measurement interval is approximately 24 hours; that is, measurements are taken at approximately the same time each day. The apparent resistivity contour map obtained using the data measured on October 13, 2021, is shown in Fig 26, and the distribution of the anomalous points is radial, which is consistent with Fig 2. Therefore, it can be determined that there are electrodes offline. The apparent resistivity contour map obtained using the data measured on October 12, 2021, is shown in Fig 27. Since there is no radial distribution of anomalous points as shown in Fig 2, it can be determined that all the electrodes are online at this time. Therefore, the electrode disconnection occurs after this measurement. There are 187 measured points in the apparent resistivity profile in Fig 26, including 49 anomalous points, and the original data integrity is 73.8%. Through analysis of the data, we determine that electrodes #49 and #50 are offline, which belongs to the continuous electrode disconnection mode. The GRNN algorithm is used to process the data, and a comparison of the predicted values and true values is shown in Fig 28. The missing values in the original data are filled with the predicted values, and the obtained apparent resistivity contour map is shown in Fig 29.

(2) Discontinuous electrode disconnection mode: This case is from the Ji 17–33200 coal mining face of the #10 Mine of the Pingdingshan Tian’an Coal Industry Co., Ltd., which is located in Weidong District, Pingdingshan City, Henan Province, China. A total of 50 brass electrodes with lengths of 0.25 m and diameters of 0.01 m are laid in the roadway at one side of the Ji 17–33200 coal mining face, and electrodes #6 to #49 are located in the effective monitoring zone. They are laid at the junction of the coal seam and its floor along the strike direction, and the electrode spacing is 10 m. To ensure good coupling between the electrodes and the coal seam floor, the electrodes are completely inserted into the coal seam floor by hammering, and the gaps are filled with yellow mud. The array type is also set as a Wenner α array. The measurement interval is approximately 24 hours; that is, measurements are taken at approximately the same time each day. The apparent resistivity contour map obtained using the data measured on September 15, 2022, is shown in Fig 30, and the distribution of the anomalous points is radial, which is consistent with Fig 2. Therefore, it can be determined that there are electrodes offline. The apparent resistivity contour map obtained using the data measured on September 14, 2022, is shown in Fig 31. Since there is no radial distribution of anomalous points as shown in Fig 2, it can be determined that all the electrodes are online at this time. Therefore, the electrode disconnection occurs after this measurement. There are 301 measured points in the apparent resistivity profile in Fig 30, including 57 anomalous points, and the original data integrity is 81.06%. Through analysis of the data, we determine that electrodes #17 and #19 are offline, which belongs to the discontinuous electrode disconnection mode. The GRNN algorithm is used to process the data, and a comparison of the predicted values and true values is shown in Fig 32. The missing values in the original data are filled with the predicted values, and the obtained apparent resistivity contour map is shown in Fig 33.

## Discussion

Firstly, the results of the experiments conducted in the water tank are quantitatively analyzed. The evaluation indexes used are the mean absolute percentage error (MAPE) and the root mean square error (RMSE), which are calculated as follows:


$$MAPE=\frac{\sum_{i=1}^{n}\left|\frac{V_{P}(i)-V_{T}(i)}{V_{T}(i)}\right|}{n}$$ (14)

$$RMSE=\sqrt{\frac{\sum_{i=1}^{n}{\left[V_{P}(i)-V_{T}(i)\right]}^{2}}{n}}$$ (15)

where $V_{P}$ is the predicted value, $V_{T}$ is the true value, and n is the number of missing values in the apparent resistivity profile.

The four groups of data in Figs 14–17 are calculated using Eq. (14), and the obtained results are presented in Table 1. Using 1-MAPE as the predicted data accuracy, when the number of offline electrodes is 1, the original data integrity is 82.96%, and the predicted data accuracy reaches 91.46%. As the number of offline electrodes increases, the original data integrity gradually decreases, and the ratio of the number of sample points in the testing set to the number of sample points in the training set gradually increases, resulting in a gradual decrease in the prediction performance. When the number of offline electrodes is 4, the original data integrity is 45.19%, and the predicted data accuracy is 76.67%. Based on quantitative analysis, since the number of sample points in the testing set is larger than that in the training set, the predicted values are no longer considered to be credible.

**Table 1:** Data evaluation indexes of the predicted data presented in Figs 14–17.

Number of offline electrodes	Number of sample points in training set (Number of normal points)	Number of sample points in test set (Number of anomalous points)	Original data integrity	RMSE	MAPE	Predicted data accuracy (1-MAPE)
**1**	112	23	82.96%	8.89	8.54%	91.46%
**2**	92	43	68.15%	9.73	13.62%	86.38%
**3**	75	60	55.56%	12.14	17.55%	82.45%
**4**	61	74	45.19%	16.60	23.33%	76.67%

Next, the results of the experiments conducted in the water tank are qualitatively analyzed. The curves of the predicted values and true values shown in Figs 14–16 are basically consistent. However, the curves of the predicted values and true values shown in Fig 17 are significantly different; this result is consistent with the results of the quantitative analysis presented earlier. Figs 18–21 are the apparent resistivity contour maps obtained by filling in the missing values in the original data with the predicted values. The low-resistivity areas in Figs 18–20 are basically consistent with that in Fig 5, which shows the location of the anomalous body in Fig 4. However, this feature does not appear in the corresponding area in Fig 21. Therefore, based on qualitative analysis, for the data with an original data integrity of 45.19%, the predicted values are no longer considered to be credible.

Since the electrode disconnection mode can be classified into continuous disconnection and discontinuous disconnection, the missing data mode can also be classified into continuous missing at specific locations and random discontinuous missing. Next, the impact on model performance of these two missing data modes is analyzed, and the data used are from Figs 11 and 23. The data evaluation indexes of the predicted data are presented in Table 2.

**Table 2:** Data evaluation indexes of the predicted data of different electrode disconnection modes.

Data	Electrode disconnection mode	Number of sample points in training set (Number of normal points)	Number of sample points in test set (Number of anomalous points)	Original data integrity	MAPE	Predicted data accuracy (1-MAPE)
**Fig 11**	Continuous	92	43	68.15%	13.62%	86.38%
**Fig 23**	Discontinuous	92	43	68.15%	11.74%	88.26%

As can be seen from Table 2, the original integrities of the data in Figs 11 and 23 are the same, but the electrode disconnection modes are different. Since the predicted accuracy of the data in Fig 23 is greater than that of the data in Fig 11, it indicates that the prediction of the missing values caused by the continuous electrode disconnection is more difficult. Therefore, the experiments conducted in the water tank mainly simulate the most extreme case of electrode disconnection; that is, many continuous electrodes are simultaneously offline.

For the actual measurement carried out in the 1295 coal mining face of the Gequan East Mine, since electrodes #49 and #50 are offline on October 13, 2021, the data measured when all the electrodes are online on October 12, 2021, is selected as the reference value for comparison. The curve of the predicted values shown in Fig 28 is basically consistent with the curve of the true values. After calculation, the MAPE is 14.82%, so the predicted data accuracy is 85.18%. Fig 29 is the apparent resistivity contour map obtained by filling in the missing values in the original data with the predicted values, and it is basically consistent with Fig 27.

Similarly, for the actual measurement carried out in the Ji 17–33200 coal mining face of the Pingdingshan #10 Mine, since electrodes #17 and #19 are offline on September 15, 2022, the data measured when all the electrodes are online on September 14, 2022, is selected as the reference value for comparison. The curve of the predicted values shown in Fig 32 is basically consistent with the curve of the true values. After calculation, the MAPE is 10.72%, so the predicted data accuracy is 89.28%. Fig 33 is the apparent resistivity contour map obtained by filling in the missing values in the original data with the predicted values, and it is basically consistent with Fig 31.

Through quantitative and qualitative analysis, it can be concluded that for the actual measurements carried out in coal mining faces, the predicted data is basically consistent with the measured data when all the electrodes are online. Therefore, when the offline electrodes cannot be maintained in time, the predicted data can basically replace the measured data. Furthermore, the verification results show that the method proposed in this paper is not only applicable to different coal mining faces but is also effective for data measured under both the continuous and discontinuous electrode disconnection modes, which proves its generalizability.

Based on the above analysis, it can be seen that there are two main factors affecting the predicted results of the GRNN model. (1) The original data integrity: as shown in Table 1, when the other parameters are the same, the greater the original data integrity is, the higher the predicted data accuracy is. (2) The electrode disconnection mode: as shown in Table 2, when the other parameters are the same, the predicted data accuracy is higher when the electrodes are discontinuously offline than when they are continuously offline.

The difference between the verification experiments conducted in the water tank and the actual measurements in the coal mining faces lies in that the offline electrodes are completely separated from the water surface in the verification experiments in the water tank, while the offline electrodes are usually not completely detached from the coal seam floor but rather have poor contact with it in the actual measurements in coal mining faces. When a completely offline electrode acts as the potential electrode, $\Delta U_{MN}$ will theoretically approach 0, which causes the corresponding measured points to appear as low-resistivity anomalous points in the apparent resistivity contour map. However, when an electrode that has poor contact with the coal seam floor acts as the potential electrode, $\Delta U_{MN}$ will not approach 0 theoretically. On the contrary, the increase in the grounding resistance results in $\rho_{a}\to \infty$, which causes the corresponding measured points to appear as high-resistivity anomalous points in the apparent resistivity contour map. Therefore, when the electrodes laid in coal mining faces are offline, the distribution of the anomalous points in the measured apparent resistivity contour map is usually not radial areas composed of both high-resistivity and low-resistivity anomalous points as shown in Fig 23. Instead, the radial areas are completely composed of high-resistivity anomalous points as shown in Fig 26 or Fig 30. Although the components of the radial areas are different, it does not affect the judgment basis of the electrode disconnection; that is, regardless of whether the radial areas are composed of high-resistivity or low-resistivity anomalous points, as long as radial areas appear, it can be determined that there are offline electrodes. In addition, since these anomalous points are all treated as missing values and do not appear in the dataset of the GRNN model, whether these points are high-resistivity or low-resistivity points does not affect the predicted results, and thus, it does not affect the reliability of the experimental results.

**Fig 34:**
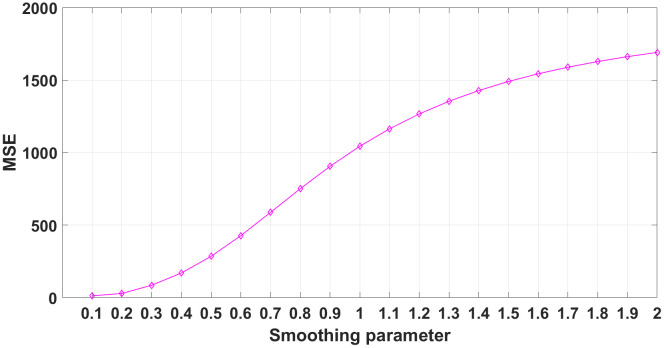
MSEs corresponding to different smoothing parameters.

Next, the sensitivity of the predicted results to different parameter choices is compared. The evaluation index used is the mean square error (MSE) between the testing set and the training set in the second cross-validation when one electrode is offline. The MSEs corresponding to different σ values are shown in Fig 34, and the MSE is the smallest when σ = 0.1. Therefore, the best smoothing parameter is determined to be 0.1.

**Fig 35:**
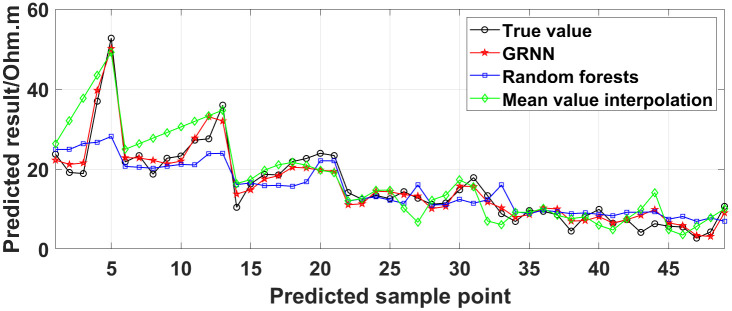
Comparison of the true values with the predicted values of the measured data of the 1295 coal mining face obtained using different methods.

Currently, the commonly used method for dealing with missing ERT values is the mean value interpolation method. In addition, the random forest algorithm is also a commonly used method. To demonstrate the superiority of the GRNN model, taking the measured data of the 1295 coal mining face as a case, we compare the true values with the predicted values obtained using the GRNN model, the random forest algorithm, and the mean value interpolation method, as shown in Fig 35. The predicted data accuracy is shown in Table 3.

**Table 3:** Predicted data accuracy of the measured data of the 1295 coal mining face of different methods.

Algorithm	Predicted data accuracy
**Mean value interpolation**	70.19%
**Random forest**	71.85%
**GRNN**	85.18%

As can be seen from Fig 35, the curve of the predicted values obtained using the GRNN model is closer to the curve of the true values than the curves of the predicted values obtained using the random forest algorithm and the mean value interpolation method. As can be seen from Table 3, for the data presented in Fig 26, the data accuracy of the predicted values obtained using the GRNN model is 13.33% and 14.99% higher than that obtained using the random forest algorithm and the mean value interpolation method, respectively. The comparison results demonstrate that the accuracy and reliability of the method proposed in this paper for the prediction of missing ERT data are superior to those of the commonly used methods.

In summary, through quantitative and qualitative analysis, for data with an original integrity greater than 55.56%, the predicted data obtained using the GRNN model is reliable, and its accuracy and reliability are better than those of the random forest algorithm and the mean value interpolation method. In the case of more extreme data loss, it is recommended that engineers immediately go to the coal mining face to maintain the electrodes.

## Conclusion

When the distribution of the anomalous points in an apparent resistivity profile is radial, it can be determined that there are electrodes offline. Electrode disconnection results in missing values in the measured data, and the missing values can be predicted using the GRNN algorithm. The verification experiments are carried out in a plastic water tank, and the results show that when the original data integrity is 82.96%, the predicted data accuracy reaches 91.46%. As the number of offline electrodes increases, the original data integrity gradually decreases, and the ratio of the number of sample points in the testing set to the number of sample points in the training set gradually increases, resulting in a gradual decrease in the prediction performance. When the original data integrity is only 55.56%, the predicted data accuracy still reaches 82.45%. However, when the original data integrity is reduced to 45.19%, the predicted data accuracy is only 76.67%, and the low-resistivity area in the corresponding apparent resistivity contour map also deviates from the location of the iron block. An actual application of our proposed method is carried out on a coal mining face. A set of data with an integrity of 73.8% is predicted. Compared with the measured data when all the electrodes are online, the accuracy of the predicted data is 85.18%. The accuracy of the data predicted using the GRNN algorithm is 14.99% higher than that of the data predicted using the commonly used mean value interpolation method. Through quantitative and qualitative analysis, we find that when the offline electrodes cannot be maintained in time, for data with an original integrity greater than 55.56%, the predicted data obtained using the GRNN algorithm is reliable.

## Supporting information

S1 Data Data used in this study. (RAR)
